# Peripapillary choroidal thickness after intravitreal ranibizumab injections in eyes with neovascular age-related macular degeneration

**DOI:** 10.1186/s12886-016-0203-7

**Published:** 2016-03-08

**Authors:** Cheolmin Yun, Jaeryung Oh, Kwang-Eon Choi, Soon-Young Hwang, Seong-Woo Kim, Kuhl Huh

**Affiliations:** Department of Ophthalmology, Korea University College of Medicine, 126-1 Anam-dong 5-ga, Sungbuk-gu, Seoul, 136-705 South Korea; Department of Biostatistics, Korea University College of Medicine, Seoul, South Korea

**Keywords:** Age-related macular degeneration, Choroidal thickness, Optical coherence tomography, Peripapillary choroidal thickness, Ranibizumab

## Abstract

**Background:**

The purpose of this study was to investigate peripapillary choroidal thickness (CT) in eyes with neovascular age-related macular degeneration (AMD) and to assess whether peripapillary CT is affected by intravitreal injection of ranibizumab (IVR) in eyes with neovascular AMD.

**Methods:**

Peripapillary and subfoveal CT were measured in spectral domain optical coherence tomography images from 39 eyes of neovascular AMD patients and 39 eyes of age-matched controls retrospectively. The patients were treated with 0.5 mg IVR monthly for 3 months and retreated as needed. Peripapillary CT at baseline, 3 months and 6 months was measured at four locations (superior, nasal, inferior and temporal areas).

**Results:**

The mean peripapillary and subfoveal baseline CTs of the eyes with neovascular AMD (153.3 ± 45.3 μm and 228.6 ± 78.6 μm) were not different from those of the controls (149.0 ± 42.3 μm and 221.4 ± 54.1 μm; *P* = 0.665 and *P* = 0.639, respectively). Subfoveal CT decreased at 3 months (213.8 ± 75.8 μm, *P* < 0.001) and 6 months (215.1 ± 72.8 μm, *P* = 0.002) following IVR treatment. Mean peripapillary CT did not show significant changes at 3 months (149.6 ± 43.8 μm, *P* = 0.156) or 6 months (150.0 ± 43.4 μm, *P* = 0.187). Subanalysis revealed that only temporal peripapillary CT decreased from baseline (167.1 ± 54.5 μm) to 3 months (159.4 ± 50.8 μm, *P* = 0.010) and was sustained at 6 months (160.6 ± 49.6, *P* = 0.026). However, superior, nasal and inferior peripapillary CT did not show significant changes after IVR.

**Conclusions:**

Changes in peripapillary CT after IVR were limited to the macular area. This result may suggest that IVR does not affect CT outside of the macula in the eyes of patients with neovascular AMD.

**Electronic supplementary material:**

The online version of this article (doi:10.1186/s12886-016-0203-7) contains supplementary material, which is available to authorized users.

## Background

Age-related macular degeneration (AMD) is a leading cause of vision loss in the elderly worldwide [1]. With the development and introduction of anti-vascular endothelial growth factor (VEGF) therapy, the functional outcomes of neovascular AMD have been markedly improved [2–4]. Ranibizumab (Lucentis; Genentech, Inc., San Francisco, CA; co-developed by Genentech, Inc and Novartis), is an FDA approved VEGF-A inhibitor that is commonly used for the treatment of AMD [5].

Previous studies have suggested that AMD may be a vascular disorder associated with altered blood flow in the choroid [6, 7]. Because abnormalities in choroidal circulation and structure are thought to be associated with the pathogenesis of AMD [8, 9], there have been many studies on choroidal thickness (CT) in AMD [10–13]. Recently, some studies have suggested that the CT in patients with neovascular AMD may be affected by intravitreal injection of ranibizumab (IVR) [14, 15]. Because VEGF is known to be associated with the maintenance of choriocapillaries and ocular blood flow, there have been concerns regarding potential undesired effects of anti-VEGF therapy on the choroid [16, 17]. However, recent studies found that CT changes following IVR treatment for neovascular AMD were limited to the choroid of the macular region [14, 15]. It is not certain whether CT outside the macula is affected by IVR, and the effect of anti-VEGF therapy on CT of normal choroid without overlying retinal pathology remains unclear [18].

The choroid is a highly vascular tissue of the eye that supplies blood to the outer retina, retinal pigment epithelium (RPE), and possibly the optic nerve head [19]. The choroid is supplied by various ciliary arteries that originate from the ophthalmic arteries [20]. The branches of the posterior ciliary artery (PCA) consist of long PCAs and short PCAs [20]. The posterior choriocapillaries in the peripapillary and submacular region are supplied by the short PCAs. These arteries have strictly segmental blood flow without anastomosis and supply a well-defined sector of the choroid [21]. The medial PCA supplies the nasal choroid, while the lateral PCA supplies the area of the choroid not supplied by the medial PCA [20]. Therefore, blood flow to the nasal peripapillary choroid could differ from the flow to the temporal peripapillary and subfoveal choroid. Recently, several studies on the choroid outside of the macula in retinal disease have focused on the peripapillary choroid and the nasal peripapillary choroid represented the choroid outside of macula [22–24]. We hypothesized that the change in CT after IVR would differ between the macula and areas outside of the macula. We also investigated the peripapillary and subfoveal CT in neovascular AMD and the change in CT after IVR treatment.

## Methods

The Institutional Review Board of Korea University Medical center approved this study (IRB No. ED14121), and all research and data collection was conducted in accordance with the tenets of the Declaration of Helsinki. We performed a retrospective case–control study of patients 50 years of age or older who were diagnosed with neovascular AMD between February 2011 and March 2013 at Korea University Medical Center. We included patients who underwent IVR for neovascular AMD. Age-matched controls were selected from the spectral domain optical coherence tomography (SD-OCT) database based on classification of normal eyes. Patients with a primary diagnosis of AMD, a history of laser photocoagulation of the macula, serous detachment of the macula, rhegmatogenous or tractional retinal detachment, high myopia, tapetoretinal degeneration, optic disc abnormalities such as glaucoma, beta zone peripapillary atrophy, or neurologic deficits were excluded from the control group.

Neovascular AMD was diagnosed in patients who were older than 50 and showed exudative changes from choroidal neovascularization (CNV) on fluorescein angiography (FA) and SD-OCT. This study excluded cases of polypoidal choroidal vasculopathy (PCV). PCV was diagnosed based on the presence of CNV on FA with characteristic findings on SD-OCT and if any one of the following criteria were met: 1) branching vascular networks underneath the RPE and focal hyperfluorescent polyps were present on indocyanine green angiography, 2) recurrent hemorrhagic or serous pigmented epithelial detachments were detected, or 3) the existence of an elevated reddish-orange lesion protruding from the choroid on fundus examination [25–28]. Neovascular lesions were classified into type 1 (sub-RPE), type 2 (subretinal) or type 3 (intraretinal) according to the MPS criteria and to the guidelines provided by Freund and colleagues based on angiographic and OCT features [29–31]. Classifications were independently determined by two retina specialists (C.Y. and J.A.). Disagreements were resolved upon further review by two observers. Cases with refractive errors ≤ -6.0 diopters or axial length ≥ 26.0 mm and cases with a history of cataract surgery within the past 6 months, refractive surgery or vitreoretinal surgery were also excluded from this study. Patients with a history of glaucoma, optic nerve disorder, beta zone peripapillary atrophy, retinal or choroidal disorder including vascular disease or uveitis and previous treatment with photodynamic therapy or other intravitreal anti-VEGF injection were also excluded from this study. Images were excluded if they were found to be low quality with a signal strength indicator (Q-factor) value < 45 or if the choroidal layer was not visible due to severe subfoveal hemorrhage. Data were collected at baseline, 3 months and 6 months after IVR.

### Intravitreal injection of ranibizumab

A single surgeon (JO) performed all injections. Before intravitreal injection, local anesthesia was induced with proxymetacaine hydrochloride drops (Alcaine 0.5 %; Alcon, Fort Worth, TX, USA). After the periocular disinfection procedure, the patient was draped. With lid speculum insertion, 5 % povidone iodine was introduced on the conjunctiva for 30 s. After injection of 0.05 ml ranibizumab into the eye 3.5 mm from the limbus, antibiotic eye drops were administered. After three monthly injections of IVRs, patients were followed up every month, and retreatment was considered if any of the following changes were observed: (1) visual acuity loss with the presence of fluid in the macula detected by OCT, (2) OCT central retinal thickness (CRT) increased by at least 100 μm, (3) new macular hemorrhage, or (4) persistent fluid on OCT at least 1 month after the previous IVR [32]. Responders were defined as patients with a disappearance of subretinal fluid or macular edema or those with a decrease in CRT of more than 100 μm on OCT 3 months after IVR as compared to the initial visit. Non-responders were defined as those with no change in subretinal fluid or macular edema or with a decrease in CRT of less than 100 μm on OCT when compared to the initial visit.

### Spectral domain optical coherence tomography

This study used a SD-OCT (3D OCT-1000 Mark II, software version 6.21; Topcon Corp., Tokyo, Japan) with a wavelength of 840 nm, a horizontal resolution of less than 20 μm, and an axial resolution of up to 5 μm. Choroidal mode was used to capture choroidal layer images. Horizontal 6-mm line-scans centered on the fovea and circle scans in a 3.4-mm zone around the optic disc were obtained. The line scan and circle scan consisted of 1024 A-scans, and circle scan images were scanned four times. All images were averaged to improve the signal-to-noise ratio.

### Measurement of peripapillary choroidal thickness

**Fig. 1:**
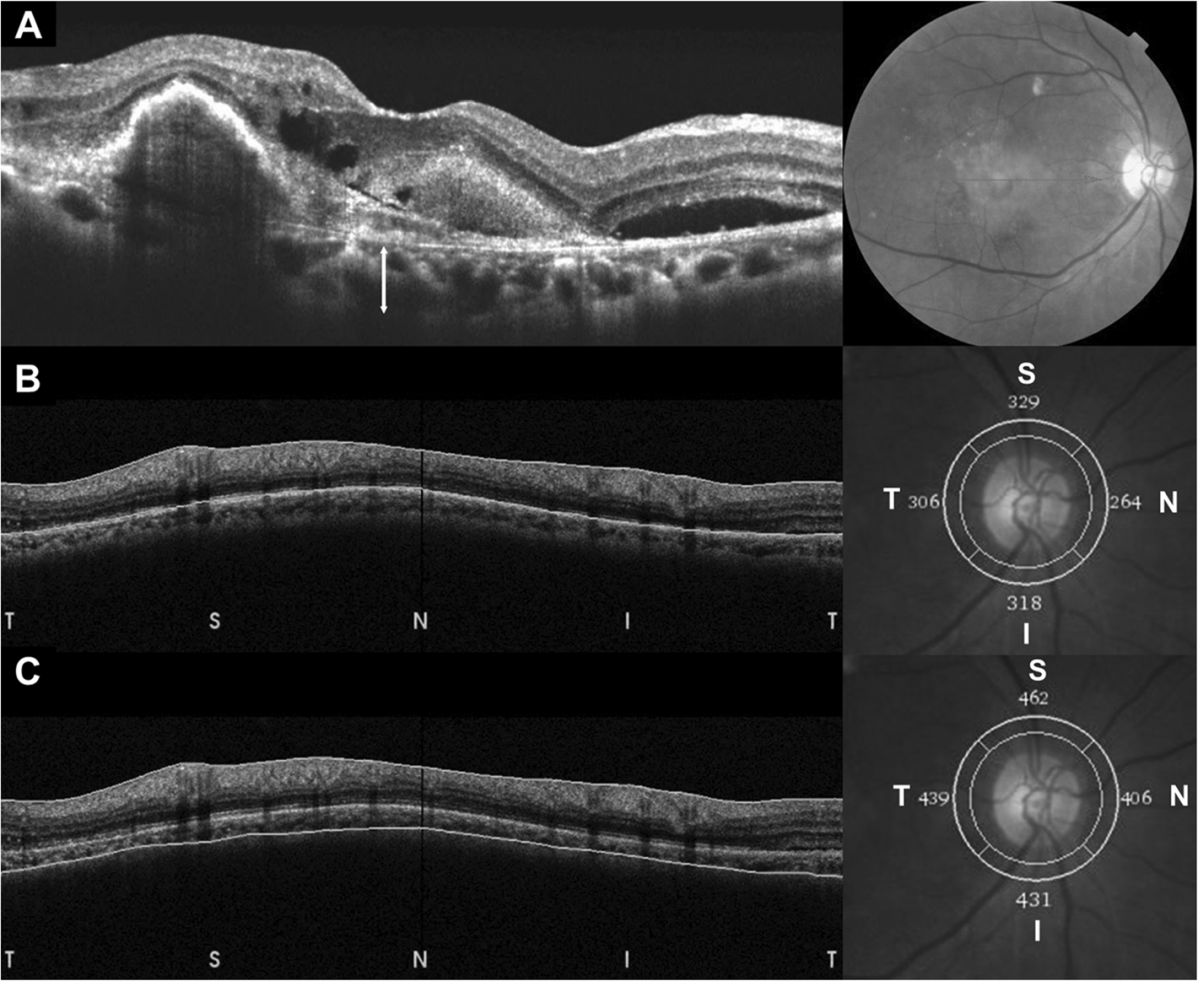
Measurement of subfoveal and peripapillary choroidal thickness (CT). Subfoveal CT (white arrow) was measured perpendicularly from the outer surface of the line corresponding to the retinal pigment epithelium (RPE) to the inner chorioscleral junction (**a**). A 360°, 3.4-mm-diameter circle scan provided retinal thickness (RT) and retinal nerve fiber layer (RNFL) thickness from four sectors around the optic disc. The thickness value of each sector represents the mean thickness of the layers within each sector (**b**). The RT of each sector was determined between two segmentation lines of the internal limiting membrane (ILM) and RPE. The segmentation line of the RPE was then modified to the chorioscleral junction by the examiner using the modification tool in the optical coherence tomography image viewer program (**c**). Chorioretinal thickness between the ILM and chorioscleral junction was obtained, and CT was calculated by subtracting the RT from the chorioretinal thickness of each sector. T, temporal; S, superior; I, inferior; N, nasal

Peripapillary CT was measured in a 3.4-mm diameter circle scan performed around the optic disc. The circle scan was performed using a standard protocol for retinal nerve fiber layer (RNFL) assessment, and the retinal thickness (RT) and RNFL thickness was assessed in four sectors (superior, nasal, inferior and temporal) around the disc. The thickness value represents the mean thickness of the layers within each sector. Peripapillary RT and RNFL thickness were obtained through automatic segmentation using the OCT viewer software after confirmation of appropriately placed segmentation lines. In cases with abnormal segmentation of the RPE or NFL due to drusen (*n* = 1) or posterior hyaloid membranes (*n* = 3), the segmentation errors were modified. Peripapillary CT was measured using a previously reported method [22, 24, 33]. Briefly, the modification tool in the SD-OCT image viewer program was used to move the segmentation line indicating RPE to the chorioscleral junction. With this modification, we obtained the chorioretinal thickness between the ILM and chorioscleral junction. Peripapillary CT was calculated by subtracting RT from the chorioretinal thickness in each sector (Fig. 1). All measurements were performed by two independent blinded examiners (CY and KC), and the mean value from the two observers was used for analysis.

### Measurement of subfoveal choroidal thickness

Choroidal layer images of the macula were obtained using choroidal mode within the OCT with a 6-mm line scan image centered on the fovea [12, 34]. CT measurement was performed manually with a caliper tool built in the image viewer program of SD-OCT. CT was defined as the length between the outer surface of a hyper-reflective line of the RPE and the inner margin of the chorioscleral junction perpendicularly (Fig. 1). All measurements were performed by two independent, blinded examiners (CY and KC). The mean value from the two observers was used for analysis.

### Statistical methods

Controls were selected from the hospital OCT database and frequency-matched to the age distribution of AMD cases with a statistician’s assistance. If a patient had neovascular AMD in both eyes, the right eye was selected for analysis, and age matching was based on the statistician’s discretion.

A Kolmogorov-Smirnov test was performed to determine whether the variables demonstrated a normal distribution. The student *t*-test, chi-square test and Fisher’s exact test were used to compare the parameters between AMD patients and the controls. A paired *t*-test was used to analyze the difference between the peripapillary CT, RT and RNFL thickness means in each sector and the macular CT during each visit. The Pearson’s correlation test was used to analyze the relationships between parameters.

By comparing CT before and after IVR, the proportional change in CT was calculated as the difference in CT (CT at 3 months – CT at baseline) divided by CT at baseline. The control group parameters included mean refractive error, RT, RNFL thickness and CT of the right eye. Statistical analyses were performed using SPSS software version 20.0 for Windows (IBM Corp., Armonk, NY, USA). Results were considered statistically significant at *P* values < 0.05.

## Results

After reviewing the medical records of 79 patients who were diagnosed with neovascular AMD, we excluded 23 cases of PCV, two cases of high myopia, one case of history of vitreoretinal surgery, three cases of low quality image, six cases of glaucoma, five cases of beta zone peripapillary atrophy. In a total of 39 eyes of 39 patients with neovascular AMD and an equal number of controls eyes were included in this study. Mean age at diagnosis in the neovascular AMD group was not different from that of the control group (Table 1). There were no differences in sex or lens status between the two groups. The mean number of IVRs within the 6-month period was 4.4 ± 0.9 (range, 3–6). Mean BCVA (LogMAR) of the 39 patients was 0.73 ± 0.45 at baseline, 0.50 ± 0.38 at 3 months, and 0.51 ± 0.44 at 6 months. Mean BCVA values at 3 months and 6 months were significantly improved when compared to baseline (all, *P* < 0.001). At baseline, the neovascular lesions were categorized as type 2 (35 eyes, 89.7 %) and type 3 (4 eyes, 10.3 %).

**Table 1:** Baseline characteristics of patients with age-related macular degeneration and age-matched controls

	AMD group	Control group	*P* value
	(*n* = 39)	(*n* = 39)	
Mean age at diagnosis (years)	68.8 ± 6.5	68.3 ± 6.5	0.755*
Sex (M : F)	18 : 21	16 : 23	0.648**
Lens (Phakia : Pseudophakia)	37 : 2	35 : 4	0.675**
Refractive error (D)^a^	0.46 ± 1.43	0.34 ± 1.64	0.734*
Type of neovascularization, eyes (%)			
Type 2	35 (89.7 %)		
Type 3	4 (10.3 %)		

Continuous variables are expressed as mean ± standard deviation

*P value based on student *t*-test

**P value based on Chi-square test or Fisher’s exact test for categorical variables

^a^Refractive errors were obtained from 37 eyes in the AMD group and 35 eyes in the control group, which included all eyes except two eyes exhibiting pseudophakia in the AMD group and four eyes exhibiting pseudophakia in the control group

*AMD* age-related macular degeneration, *M* Male, *F* female, *D* diopters

### Comparison of baseline choroidal thickness in AMD eyes and controls

The mean peripapillary CT of the eyes in the AMD group was not significantly different from that of the control group (Table 2). In the control group, the mean peripapillary CT correlated with age (*r* = - 0.415, *P* = 0.009), but the mean peripapillary CT of the AMD group did not correlate with age (*r* = - 0.169, *P* = 0.303).

**Table 2:** Comparison of choroidal thickness between patients with neovascular age-related macular degeneration and age-matched controls

	AMD group	Control group	*P* value*
	(*n* = 39)	(*n* = 39)	
Subfoveal CT (μm)	228.6 ± 78.6	224.2 ± 54.7	0.775
Peripapillary CT (μm)			
Mean	153.2 ± 45.3	148.2 ± 38.8	0.600
Superior	165.2 ± 49.5	160.3 ± 42.6	0.637
Nasal	153.2 ± 43.0	148.3 ± 36.5	0.591
Inferior	127.3 ± 40.0	122.7 ± 32.5	0.577
Temporal	167.1 ± 54.5	161.4 ± 49.8	0.635

Continuous variables are expressed as mean ± standard deviation

*Comparison of baseline parameters between the control group and AMD group, *P* value was based on the student *t*-test

*AMD* age-related macular degeneration, *CT* choroidal thickness

The mean subfoveal CT of the AMD group was not significantly different from that of the control group (Table 2). In the control group, subfoveal CT correlated with age (*r* = - 0.386, *P* = 0.015), but subfoveal CT in the AMD group did not correlate with age (*r* = - 0.162, *P* = 0.324).

### Choroidal thickness after intravitreal injection of ranibizumab

**Fig. 2:**
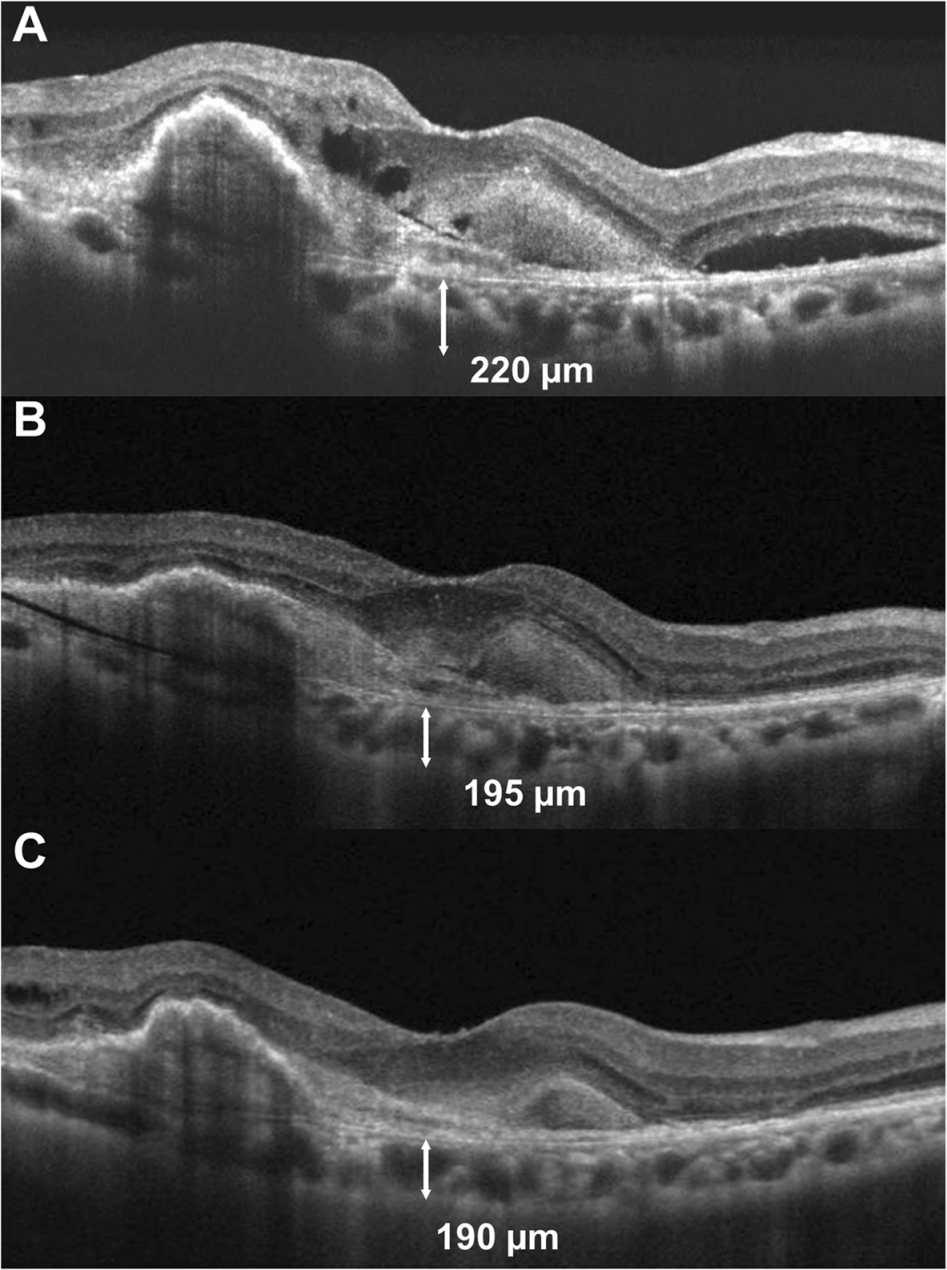
Changes in subfoveal choroidal thickness (SFCT) in a 74-year-old woman with neovascular age-related macular degeneration. SD-OCT image at baseline (**a**) showed subretinal fluid accumulation in the macula. After three consecutive monthly injections of ranibizumab, the subretinal fluid disappeared, and the SFCT decreased as compared with the baseline (**b**). At 6 months, the SFCT was still lower compared with baseline (**c**)

**Fig. 3:**
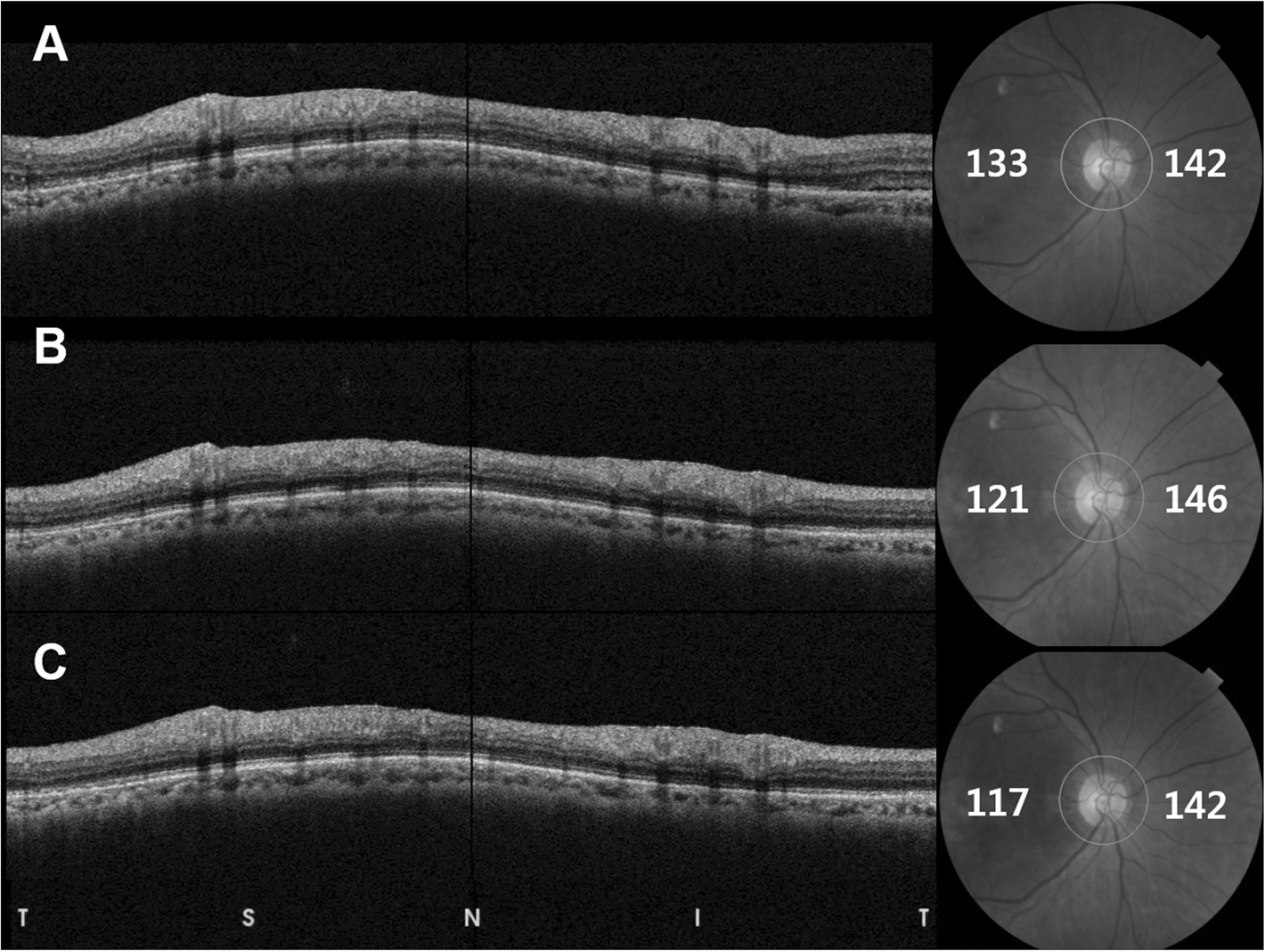
Changes in peripapillary choroidal thickness (PCT) of the patient shown in Fig. 2. Peripapillary circle scans at baseline (**a**), 3 months (**b**) and 6 months after the intravitreal injection of ranibizumab (**c**). Nasal PCT at baseline (142 μm) was not changed at 3 months (146 μm) and 6 months (142 μm), while temporal PCT at baseline (133 μm) decreased at 3 months (121 μm) and 6 months (117 μm). T, temporal; S, superior; I, inferior; N, nasal

The mean peripapillary CT at baseline in the AMD group decreased after IVR, but this was not a statistically significant difference at 3 months or 6 months (Table 3). Among the four sectors of the peripapillary area, only the temporal peripapillary CT decreased at both 3 and 6 months, while other areas showed no significant changes. Mean subfoveal CT at baseline in the AMD group was significantly reduced at 3 months and at 6 months (Table 3) (Figs. 2 and 3). The difference in value or proportion of subfoveal and mean peripapillary CT at 6 months was not associated with the number of IVRs received (Pearson’s correlation test, all, *P* > 0.05). Mean subfoveal CT and peripapillary CT at both 3 and 6 months showed no significant differences when compared with the baseline values of the normal controls. An additional file shows this in more detail [Additional file 1].

**Table 3:** Changes in choroidal thickness, retinal thickness and retinal nerve fiber layer thickness during ranibizumab treatment

	Baseline	3 months	*P* value*	6 months	*P* value*
Subfoveal CT (μm)	228.6 ± 78.6	213.8 ± 75.5	<0.001	214.1 ± 72.8	<0.001
Peripapillary CT (μm)					
Mean	153.2 ± 45.3	149.6 ± 43.8	0.156	150.0 ± 43.4	0.187
Superior	165.2 ± 49.5	162.2 ± 48.7	0.252	162.6 ± 47.8	0.330
Nasal	153.2 ± 43.0	151.6 ± 43.3	0.532	150.8 ± 42.7	0.378
Inferior	127.3 ± 40.0	125.1 ± 38.3	0.321	125.9 ± 37.5	0.555
Temporal	167.1 ± 54.5	159.4 ± 50.8	0.010	160.6 ± 49.6	0.011
Central RT (μm)	300.4 ± 93.0	233.6 ± 62.0	0.001	232.9 ± 61.9	0.001
Peripapillary RT (μm)					
Mean	282.8 ± 19.7	279.9 ± 22.1	0.009	279.8 ± 20.2	0.033
Superior	303.0 ± 28.3	301.6 ± 30.1	0.169	300.7 ± 26.9	0.171
Nasal	250.4 ± 22.1	251.0 ± 26.7	0.732	251.0 ± 22.9	0.812
Inferior	300.4 ± 22.0	300.2 ± 24.8	0.829	298.2 ± 22.7	0.246
Temporal	276.8 ± 21.2	267.0 ± 20.1	<0.001	269.3 ± 21.9	0.015
RNFL thickness (μm)					
Mean	111.7 ± 11.4	112.9 ± 10.6	0.666	113.1 ± 11.9	0.611
Superior	130.4 ± 17.5	131.8 ± 16.3	0.736	134.0 ± 16.4	0.373
Nasal	89.3 ± 15.8	92.2 ± 18.9	0.478	90.4 ± 19.3	0.790
Inferior	135.1 ± 16.9	137.9 ± 17.0	0.439	138.6 ± 18.9	0.350
Temporal	92.1 ± 16.2	89.4 ± 13.4	0.379	89.4 ± 16.0	0.385

Continuous variables are expressed as mean ± standard deviation

*Comparison of the parameters in each AMD group visit; *P* value was based on the paired *t*-test

*CT* choroidal thickness, *RT* retinal thickness, *RNFL* retinal nerve fiber layer

By neovascularization type, the type 2 group showed decreased subfoveal CT at both 3 and 6 months (all, *P* < 0.05), while the mean peripapillary CT showed no changes (all, *P* > 0.05) (Table 4). The type 3 group showed a tendency toward decreased subfoveal CT, but this was not seen in mean peripapillary CT. Because of the small number of type 3 cases, the statistical power was not available.

**Table 4:** Changes in choroidal thickness during ranibizumab treatment according to neovascularization type

		Baseline	3 months	*P* value*	6 months	*P* value*
Type 2	Subfoveal CT (μm)	238.7 ± 75.8	223.4 ± 72.4	<0.001	224.0 ± 68.5	0.002
(*n* = 35)	Peripapillary CT (μm)					
	Mean	157.3 ± 45.1	153.3 ± 43.1	0.154	153.4 ± 42.6	0.163
	Superior	168.8 ± 47.9	165.6 ± 46.3	0.266	165.9 ± 45.3	0.313
	Nasal	155.9 ± 43.7	154.1 ± 43.5	0.511	153.2 ± 42.4	0.340
	Inferior	131.7 ± 39.8	129.2 ± 38.1	0.285	129.9 ± 38.3	0.456
	Temporal	172.5 ± 53.6	164.4 ± 49.4	0.011	164.5 ± 49.2	0.010
Type 3	Subfoveal CT (μm)	145.0 (102.8, 171.2)	124.0 (89.5, 175.8)	N/A	117.0 (83.4, 182.5)	N/A
(*n* = 4)	Peripapillary CT (μm)					
	Mean	112.6 (89.3, 150.8)	117.0 (80.0, 152.5)	N/A	117.0 (83.8, 182.5)	N/A
	Superior	125.0 (82.5, 193.8)	129.0 (72.8, 194.3)	N/A	131.0 (75.0, 195.3)	N/A
	Nasal	132.5 (98.5, 156.0)	129.5 (91.0, 167.3)	N/A	128.0 (87.5, 173.0)	N/A
	Inferior	88.5 (83.0, 93.3)	84.5 (77.8, 104.0)	N/A	84.5 (77.8, 110.0)	N/A
	Temporal	112.5 (84.0, 161.3)	116.5 (71.5, 160.0)	N/A	117.0 (71.3, 162.8)	N/A

Continuous variables are expressed as mean ± standard deviation or median (interquartile range)

*Comparison of parameters in each AMD group visit; *P* value was based on paired *t*-test

*CT* choroidal thickness, *N/A* not applicable

Thirty eyes (76.9 %) showed treatment response and nine eyes (23.1 %) showed non-response. In univariate analysis, a decreased proportion of both subfoveal CT and temporal peripapillary CT were associated with treatment response (Table 5). In multivariate analysis, only a decreased proportion of subfoveal CT was associated with treatment response (Table 6).

**Table 5:** Univariate analysis for estimating factors associated with a 3-month response after intravitreal injection of ranibizumab

Variables	*P* value*	OR (95 % CI)
Sex (male)	0.521	1.635 (0.365–7.326)
Age (years)	0.945	1.004 (0.894–1.128)
Eye (OD)	0.237	0.400 (0.088–1.826)
Lens status (phakic eye)	1.000	N/A
Baseline subfoveal CT (μm)	0.296	0.994 (0.984–1.005)
Baseline mean peripapillary CT (μm)	0.789	0.998 (0.981–1.015)
Decreased amount of subfoveal CT (μm)	0.097	1.030 (0.995–1.066)
Decreased proportion of subfoveal CT (%)	0.007	1.124 (1.033–1.223)
Decreased amount of mean peripapillary CT (μm)	0.094	1.041 (0.993–1.092)
Decreased proportion of mean peripapillary CT (%)	0.077	1.055 (0.994–1.120)
Decreased amount of mean temporal peripapillary CT (μm)	0.061	1.043 (0.998–1.090)
Decreased proportion of mean temporal peripapillary CT (%)	0.029	1.070 (1.007–1.137)

*P value based on logistic regression analysis

*OD* oculus dexter, *CT* choroidal thickness, *N/A* not applicable

**Table 6:** Multivariate analysis for estimating factors associated with a 3-month response after intravitreal injection of ranibizumab

Variables	*P* value*	OR (95 % CI)
Sex (male)	0.511	1.879 (0.287–12.306)
Age (years)	0.602	1.038 (0.903–1.193)
Decreased proportion of subfoveal CT	0.042	1.362 (1.016–1.825)
Decreased proportion of mean temporal peripapillary CT	0.166	0.861 (0.696–1.064)

*P value based on logistic regression analysis

*CT* choroidal thickness

### Retinal thickness and retinal nerve fiber layer thickness

CRT and temporal peripapillary RT of the AMD group decreased at 3 months and at 6 months when compared with the baseline values, while the mean RNFL thickness showed no significant change 3 months or 6 months after IVR (Table 3).

### Interobserver reliability

Interobserver reliability was assessed by analysis of intraclass correlation coefficients. The intraclass correlation coefficients ranged from 0.940 to 0.984. An additional file shows this in more detail [Additional file 2].

## Discussion

In this study, macular and peripapillary CT in the AMD group did not differ from those in the control group at baseline. The macular CT results in AMD patients and normal controls are consistent with those found in previous studies, which revealed no significant differences between AMD eyes and normal eyes [11–13]. Because it has been suggested that AMD is a vascular disorder associated with altered blood flow in the choroid [6, 7], we wondered if there were regional differences in CT between the macula and outside of the macula. However, we found no such difference in CT between the macula and outside of the macula in the neovascular AMD and control groups.

Peripapillary CT outside of the macula did not change after IVR at 6 months. These findings differed from the subfoveal choroid results. In this study, both subfoveal CT and temporal peripapillary CT decreased after three monthly IVRs, and the reduction in CT was sustained for 6 months. CT outside of the macula, including superior, nasal and inferior peripapillary CT, was not affected by IVR. Even though there was a trend toward a reduction in PCT in all sectors, these differences were not statistically significant. This may indicate that ranibizumab affects the choroid under and around the macula but not the choroid outside of the macula. We did not investigate the change in CT in AMD eyes without treatment. Therefore, the results of our study could not confirm if choroidal thinning of the subfoveal area is directly induced by the IVR or by the disease course of AMD. However, the results of the current study may suggest that the CT outside of the macula is not involved in the change in CT after IVR in eyes with AMD.

In the current study, subfoveal CT decreased after IVR, a result consistent with previous studies [14, 15]. Yamazaki et al. suggested that IVR may have a pharmacologic effect on the choroid underneath the CNV lesion [15]. Changes in CT associated with IVR may be explained by two possible mechanisms. First, decreased intraocular VEGF after IVR might affect the CT. VEGF is known to be associated with vasculogenesis and blood vessel hyperpermeability [35–37]. Hypoxia and ischemia of RPE from inadequate choroidal perfusion might produce VEGF, which may lead to neovascular AMD [38]. Both the subfoveal choroid and the peripapillary choroid may be affected by increased VEGF. Although the natural course of CT in neovascular AMD is unknown, vasodilatation may be expected in the choroid of neovascular AMD eyes, as VEGF is associated with vascular permeability [36, 37]. After a decrease in intraocular VEGF levels due to IVR, changes in vascular permeability and CT in both the macular and peripapillary region may follow. In this study, the changes in macular and temporal peripapillary CT were significant while CT outside of macula showed no significant changes. This might suggest that IVR mainly affects the abnormal CNV and the hyperpermeability associated with the CNV under and around the macula. In addition, vasoconstriction of the choroid after IVR may affect CT. Although the effect of VEGF on choroidal structure, thickness and large vessels is not clearly defined, VEGF is known to be essential for the development of the choroid and maintenance of the choriocapillaries [39, 40]. Furthermore, the vasoconstrictive effect of ranibizumab might affect the choroidal vessels and thickness [41, 42]. In this study, a significant change in CT was observed only in the macular and temporal peripapillary areas. Thus, even though we cannot exclude the vasoconstrictive effect of IVR on the macula, IVR does not result in a change of CT outside of the macula.

In this study, decreased proportions of subfoveal and temporal peripapillary CT were associated with treatment response after three monthly doses of ranibizumab. However, in multivariate analysis, a decreased proportion of subfoveal CT was a factor associated with treatment response, which might result from the positive correlation between a decreased proportion of subfoveal CT and temporal peripapillary CT. This may suggest that decreased hyperpermeability or vasoconstriction and regression of CNV after IVR change the choroid under and around the macula. However, we cannot conclude whether decreased CT is a preceding factor associated with treatment response or if decreased CT is a result of treatment response, as the decreased ratio of CT is not a preoperative finding but rather a parameter that is calculated from CT before and after IVR.

However, the observed decrease in CT is insufficient to limit the clinical use of ranibizumab. In the present study, although macular and temporal peripapillary CT were decreased at 3 and 6 months after IVR when compared to the initial CT, the reduction in CT was relatively small, and was not associated with a loss of BCVA. In addition, CT after IVR at 3 and 6 months was not different from that of the normal controls. In addition, the non-significant changes in CT outside of the macula suggest that the effect of IVRs outside of the macula is minimal. IVRs decreased temporal peripapillary RT, but may be associated with a decrease in CRT. The RT of other peripapillary areas and RNFL thickness were not affected by IVRs.

In the control group, the subfoveal CT and peripapillary CT showed a negative relationship with age. Previous studies have demonstrated that the subfoveal CT of normal controls and patients with dry AMD are correlated with age, but the eyes with neovascular AMD demonstrated a weaker correlation between subfoveal CT and age [13, 34]. The peripapillary CT was also related to age and had a negative correlation with the results from our previously reported study [33]. In this study, the normal control group showed a negative correlation between subfoveal and peripapillary CT and age, but the neovascular AMD group showed no correlation between subfoveal and peripapillary CT and age. This absent relationship between subfoveal CT and age might suggest an alteration in the choroid of the macula in neovascular AMD and may provide additional insight into the pathogenesis of neovascular AMD [15].

This study has several limitations. First, because it is a retrospective study with short-term results and contains a small number of cases, our study may have limited generalizability. We could not see a short-term change in CT 1 month after IVR, and it is insufficient to draw conclusions on the long-term effect of IVR on the choroid based on the short follow-up period. Further studies with larger sample sizes and longer study periods are needed. Second, because of the retrospective nature of this study, diurnal variation of CT was not considered. Mean diurnal variation of subfoveal CT in healthy individuals is about 33.7 μm and the diurnal variation of the peripapillary CT remains unclear [43]. The changes in the mean macular and peripapillary CT in this study were relatively small, and the changes in macular CT were less than those expected from diurnal variation. Although most patients visited the outpatient clinic during the day, this study did not adjust for diurnal changes in CT. Third, we could not obtain information about refractive error and axial length in all patients. Two patients in the AMD group and four patients in the control group with pseudophakia had no information available on refractive error, although the axial length in all of these patients was less than 26.0 mm. Because axial length and refractive error are known to be associated with subfoveal CT and peripapillary CT, the lack of this information in the analysis is a shortcoming of this study. Fourth, although CT measurements were performed by two independent examiners, the method was manual as no automated method exists. Fifth, we only matched patients by age, but not by sex. Although there were no differences in these characteristics between the two patient groups, the results may still be affected. Sixth, we only measured CT of the peripapillary or macular region. It could not represent CT beyond the posterior pole. Finally, because the treatment was based on monthly dosing and a PRN schedule, the number of injections received at 6 months differed among patients. However, there was no significant correlation between the difference in subfoveal or peripapillary CT at 6 months and the number of injections.

## Conclusions

The mean peripapillary CT of the AMD group was not significantly different from that of the age-matched controls. Apart from the CT in the macular area, the mean peripapillary CT and peripapillary CT outside of the macula did not change 6 months after IVR. This result may suggest that the change in CT after IVR may be limited to the macula in eyes with neovascular AMD.

## Additional files

Additional file 1: Comparison of choroidal thickness between neovascular AMD eyes at 3 and 6 months and normal controls. Mean subfoveal CT and peripapillary CT at both 3 and 6 months showed no significant differences when compared with the baseline values of the normal controls. (PDF 11 kb) Additional file 2: Interobserver measurement reliability of the choroidal thickness. Interobserver reliability was assessed by analysis of intraclass correlation coefficients. The intraclass correlation coefficients indicated good agreement. (PDF 60 kb)

## Abbreviations

CT: Choroidal thickness
AMD: Age-related macular degeneration
IVR: Intravitreal ranibizumab injections
VEGF: Vascular endothelial growth factor
RPE: Retinal pigment epithelium
PCA: Posterior ciliary artery
OCT: Optical coherence tomography
CRT: Central retinal thickness
RNFL: Retinal nerve fiber layer
RT: Retinal thickness
