# Supplementary material for: Peripapillary choroidal thickness after intravitreal ranibizumab injections in eyes with neovascular age-related macular degeneration
Source: BMC Ophthalmol. 2016 Mar 8;16:25. doi: 10.1186/s12886-016-0203-7 (PMC4782363; doi:10.1186/s12886-016-0203-7)
Supplement: Additional file 1: — Comparison of choroidal thickness between neovascular AMD eyes at 3 and 6 months and normal controls. Mean subfoveal CT and peripapillary CT at both 3 and 6 months showed no significant differences when compared with the baseline values of the normal controls. (PDF 11 kb) [file 12886_2016_203_MOESM1_ESM.pdf]

Additional table 1. Comparison of choroidal thickness between neovascular AMD eyes at 3 and 6 months and normal controls.

	Control group	AMD group at 3 months	*P* value*	AMD group at 6 months	*P* value*
Subfoveal CT (µm)	224.2 ± 54.7	213.8 ± 75.5	0.489	214.1 ± 72.8	0.493
Peripapillary CT (µm)					
Mean	148.2 ± 38.8	149.6 ± 43.8	0.882	150.0 ± 43.4	0.869
Superior	160.3 ± 42.6	162.2 ± 48.7	0.857	162.6 ± 47.8	0.824
Nasal	148.3 ± 36.5	151.6 ± 43.3	0.719	150.8 ± 42.7	0.781
Inferior	122.7 ± 32.5	125.1 ± 38.3	0.765	125.9 ± 37.5	0.694
Temporal	161.4 ± 49.8	159.4 ± 50.8	0.859	160.6 ± 49.6	0.872

Continuous variables are expressed as mean ± standard deviation.

*Comparison of parameters between the control group and AMD group, *P* value was based on student t-test

CT, choroidal thickness
