# Supplementary material for: Peripapillary choroidal thickness after intravitreal ranibizumab injections in eyes with neovascular age-related macular degeneration
Source: BMC Ophthalmol. 2016 Mar 8;16:25. doi: 10.1186/s12886-016-0203-7 (PMC4782363; doi:10.1186/s12886-016-0203-7)
Supplement: Additional file 2: — Interobserver measurement reliability of the choroidal thickness. Interobserver reliability was assessed by analysis of intraclass correlation coefficients. The intraclass correlation coefficients indicated good agreement. (PDF 60 kb) [file 12886_2016_203_MOESM2_ESM.pdf]

Additional table 2. Interobserver measurement reliability of the choroidal thickness.

	Correlation coefficient	95% confidence interval
Subfoveal CT	0.963	0.943 – 0.976
Peripapillary CT		
Supeior	0.952	0.937 – 0.965
Nasal	0.964	0.951 – 0.976
Inferior	0.940	0.925 – 0.954
Temporal	0.984	0.971 – 0.996

CT, choroidal thickness.
